# The experiences and needs of couples affected by prostate cancer aged 65 and under: a qualitative study

**DOI:** 10.1007/s11764-020-00936-1

**Published:** 2020-09-24

**Authors:** Nicole Collaço, Richard Wagland, Obrey Alexis, Anna Gavin, Adam Glaser, Eila K. Watson

**Affiliations:** 1 Faculty of Health Sciences, University of Southampton, Southampton, S017 1BJ UK; 2 Faculty of Health and Life Sciences, Oxford Brookes University, Jack Straws Lane, Oxford, OX3 0FL UK; 3 Northern Ireland Cancer Registry Centre for Public Health, School of Medicine, Dentistry and Biomedical Sciences, Queen’s University, Belfast, BT12 6BA UK; 4 Leeds Institute of Cancer and Pathology, Faculty of Medicine and Health, University of Leeds, Worsley Building, Leeds, LS2 9NL UK

**Keywords:** Prostate cancer, Couples, Dyad, Partner, Qualitative, LAPCD

## Abstract

**Purpose:**

Prostate Cancer (PCa) is often considered to be an illness affecting older men, however the prevalence in younger men (<=65 years) is rising. Diagnosis and treatment for PCa can have a significant impact on the lives of both the man with PCa and his partner. This study explored the experiences and needs of younger men and their partners affected by PCa. The findings will be used to inform service provision and develop interventions appropriate to need.

**Methods:**

Participants were recruited from respondents to a national PROMS study (Life After Prostate Cancer Diagnosis (LAPCD), who indicated on completed questionnaires their willingness to be interviewed. Semi-structured telephone interviews were conducted with twenty-eight couples, separately (56 participants). Data were analysed using the Framework Method.

**Results:**

Following the diagnosis of PCa, couples’ experienced changes in their intimate relationships, parental/familial roles, work and finances, and social connections and activities. Couples adopted a range of strategies and behaviours to help their adjustment to PCa, such as communicating with each other, distancing, distraction, and adopting a positive mindset towards PCa. This, in turn, influenced how their identity as a couple evolved.

**Conclusions:**

Following a diagnosis of PCa, the identity of couples are continually evolving. It is important that these couples are provided with the appropriate information, support and resources to help them transition along the cancer pathway.

**Implications for Cancer Survivors:**

Key areas of support identified for younger couples include: 1) couple focused support programme to foster relationship strategies/behaviours that facilitate couple adjustment; 2) age-specific support, e.g. ‘buddying systems’ connecting younger couples affected by PCa and providing them with tailored information (written/online/app).

**Electronic supplementary material:**

The online version of this article (10.1007/s11764-020-00936-1) contains supplementary material, which is available to authorized users.

## Background

An estimated 1.3 million men worldwide were diagnosed with prostate cancer (PCa) in 2018 [1]. PCa is often considered an illness of older men, with about two-thirds of men diagnosed aged over 65. However, the number of younger men aged ≤ 65 diagnosed with PCa has increased nearly sixfold over the past two decades [2]. At least some of this rise in incidence is attributed to the increase in the use of PSA testing [2]. Younger men affected by PCa exhibit greater unmet psychological needs than the general population of men with PCa [3, 4].

PCa and the side effects of treatment (e.g., incontinence, erectile dysfunction, fatigue, hot flushes) impact upon the lives of both the man with PCa and his intimate partner [5]. Literature on relationships and cancer demonstrate the influence that members of a couple have on each other and that what one person experiences will impact on the other person in the dyad [6, 7]. The symptoms that men experience may disrupt the dynamics within the couple by impacting upon their ability to work, their financial situation, and their children [8], in addition to challenges posed in maintaining intimacy in the relationship [9].

Whilst a number of previous studies have explored the impact of PCa on couples, very few of these have focussed on the experiences of younger couples (where the man is ≤ 65) [10]. Findings from one qualitative study which examined the experiences of men with PCa and their partners according to their life stage cohort—(50–64), (65–74), and (75–84)—indicated that adjustment to PCa is more difficult for younger couples (aged 50–64) [9]. Younger couples in this study described feeling ‘out of sync’ with other couples of their age group who did not have cancer. Younger couples also perceived that being diagnosed at an older age would have been easier due to possibly being more financially stable and secure in other areas of their lives. Further understanding is needed of the experiences of younger couples to clearly identify their supportive care needs and understand how services can be better tailored to this increasing age cohort.

We have previously published a core theme from the findings of this study associated with the challenges faced by younger couples affected by PCa related to parenthood and family functioning [8].

## Methods

The Consolidated Criteria for Reporting Qualitative Research (COREQ) guidelines [11] were followed in reporting the study findings (see online resource 1).

## Ethics

The NRES approval (North East-Newcastle & North Tyneside 1. REC Reference no: 15/NE/0036) was granted for the wider LAPCD study and this sub-study.

### Recruitment and data collection

This article focuses on the findings from a qualitative sub-study conducted as part of a large UK-wide study: Life After Prostate Cancer Diagnosis (LAPCD) [12]. Participants were identified through cancer registries (England, Wales, and Northern Ireland) and through hospital activity data in Scotland. Participants were invited by their treating centre to complete a postal questionnaire. Surveys were returned to the Picker Institute Europe (PIE) who managed the data. Following the survey, PIE sent the research team contact details of a random sample of respondents that were eligible for this study: those who completed the LAPCD questionnaire, who were aged ≤ 65, and had indicated that they and their partner/spouse would be interested in taking part in a telephone interview.

Previous research on PCa distinguished ‘younger’ men with PCa using an age cut-off of 65 years [9, 13]. Therefore, in line with this, ‘younger’ in the context of men with PCa as defined in this study is age 65 and under.

A maximum variation sampling framework was developed according to treatment type, age, and survey responses to ensure a wide range of experiences were explored. Initially, a selection of at least five men from each of the main treatment types (active surveillance, surgery, radiotherapy, hormone treatment, and systemic therapy) were chosen. Men from within these treatment types were then sampled by age group (45–49, 50–54, 55–59, 60–65). We then sampled different survey responses/experiences across the four age groups. Specifically, varied responses taken into consideration included men who experienced no problems or one or more problems with erectile function and psychosexual support, ability to cope, and emotional and physical wellbeing. Whilst we aimed to include men from different ethnic backgrounds, there were only two men in the sample sent from the PIE who were not from a White ethnic group (both were invited). There were also no men who identified as gay in the sample provided and therefore the samples’ representation is limited to heterosexual couples.

An invitation pack was sent to selected participants which included an invitation letter, participant information sheet, reply slip, and prepaid envelope for both the man with PCa and his partner. Participants who returned their reply slip indicating willingness to participate were contacted. Couples in which only one partner wanted to take part were excluded. Verbal, recorded, and written consent was obtained from all participants prior to the start of the interview.

### Interview procedures

A meta-synthesis of the literature on couples affected by PCa informed the interview topic guide [10]. Open-ended questions and prompts were used to encourage couples to talk about their PCa experiences and its impact on their lives. Topics discussed included the impact on their intimate relationships, family life, work and finances, social relationships, as well as treatment and healthcare experiences.

Younger men with PCa and their partners were interviewed separately by telephone to allow each participant to talk about their experiences without the potential of their partner influencing their account. Interviews were conducted by a female researcher (NC) between August 2016 and July 2017 and were 30–60 min in duration. Transcripts were not shared with participants for comments or correction in order to keep partners’ versions confidential from each other.

## Data analysis

Interviews were transcribed verbatim, and thematic analysis using the Framework Method [14, 15] was undertaken. The Framework Method allowed for the data to be analysed at the level of the couple. Dyadic analysis drew upon the multi-perspective analysis methodology of Yosha et al [16]. This involved the creation of a table of patient and partner quotes which was adapted for the Framework Method through creating tables consisting of codes that were developed to create themes and subthemes relevant to the man with PCa and his partner. The Framework matrix was further adapted by including a column of dyadic summaries that were created based on these codes and therefore allowed analysis at the level of the couple. Word and Excel programmes were used to manage the data. Summaries also included field notes of the interviews conducted. An analytical framework based on the existing codes and categories was then applied to the rest of the dataset (dyadic summaries). The last stage involved interpreting the dyadic data and was conducted by exploring potential overlaps and contrasts between the interviews of each partner, informed by Eisikovits and Koren [17] (see online resource 2).

The extent of agreement for each theme was discussed between members of the research team (NC, EW, RW, OA), and further revisions were made accordingly. Data saturation was achieved for all themes when subsequent data provided no new categories.

## Results

### Study sample

Forty-six couples were invited to take part, of which 28 couples agreed and were interviewed (response rate = 61%). One couple was not eligible for the study as only the man with PCa expressed an interest to take part. Table 1 (see online resource 3) presents the sample demographic characteristics.

#### Evolving couple identity

An overarching theme of ‘evolving couple identity’ emerged from the data. Couple identity refers to the sense of ‘us’ or ‘we-ness’ in the relationship. Three core themes were identified: *Couple relationships—integrating/managing old and new relational dynamics*; *Work and finances: challenges, buffers, and new directions*; and *Development of social connections and impact on social activities*. The impact of PCa on younger couples led to significant changes to couples’ relationships, parenthood and family functioning, work and finances, social activities, and connections. Subsequently, these impacts triggered various *‘*engagement strategies and behaviours’ within couples’ relationships which influenced their adjustment to PCa and therefore couples’ sense of ‘we-ness’, their collective identity as a couple (see Fig. 1, online resource 4). The theme associated with parenthood and family functioning has been previously published [8]; thus the other three themes will be the focus of this article.

##### Couple relationships—integrating/managing old and new relational dynamics

Changes occurred to couples’ relationships due to the impact of PCa and side effects of treatment. Couples had to find ways to integrate and/or manage old and new relational dynamics. This, in turn, impacted upon their joint sense of themselves (couple identity). Changes in the roles of individuals within couples were commonly reported, in particular the types of support provided (‘It brought out a different side to us, and I was reliant upon her and I needed her at the time for that mental support’, Dyad 23, husband, 55–59, AS). Another couple in which the husband had advanced PCa experienced difficulties in accepting the role changes in their relationship. The side effects of treatment impacted upon their roles in that the man was unable to support his wife practically as he had previously:

‘What he has found hard, is he used to have to look after me, especially when my muscles seize and I can barely walk, and now he’s swapped roles. Every time I did something, he’d say “I’m meant to be looking after you”’ (Dyad 5, wife, 45–49, Chemo & HT, ST).

‘I was used to looking after her, but then I couldn’t look after her, and that made me more depressed. I’d do all the ironing and things like that used to hurt (wife), but I just didn’t have any energy’ (Dyad 5, husband, 50–54, Chemo & HT, ST).

Although wives often already played an active role in providing emotional and practical support to men, this role seemed to be magnified by the impact of PCa, as women often described feeling the pressure and need to be strong for their husband and family by *keeping it together*. Subsequently, this sometimes led women to neglect themselves (‘I just don’t have time to think about myself. I just make sure I look after him, after everybody and put me to the back of it’, Dyad 16, wife, 50–54, RP).

The impact of PCa and side effects of treatment triggered different *engagement strategies and behaviours* within couples’ relationships, which influenced their adjustment to PCa. Engagement strategies and behaviours pertain to how couples interacted with one another and included *relational communication*, *distancing from unfamiliarity*, *mindset towards PCa*, and *distraction,* which are highlighted throughout the core themes.

##### Relational communication

Communication between members of a couple is a pivotal engagement strategy that influences how they integrate and manage the impact of PCa, choose a direction of treatment, and express their feelings. Subsequently, how couples interact and react to one another can impact upon their identity as a couple. Wives often reported that they were the communication initiators, prompting their husbands to discuss what they were feeling with them. Some women and men attributed not being open about their feelings to gender norms: ‘He’s a man, and he wouldn’t talk, but I insist we talk. […] I always make sure we talk’ (Dyad 6, wife, 55–59, RP & EBR).

Communication within couples became particularly challenging regarding sexual functioning and wives often avoided such conversations not wishing to upset their husband or cause further worry and protect the couple unit from further distress, (‘I didn’t tell him I was frustrated, I didn’t want him to feel embarrassed or pressured’, Dyad 24, partner, 50–54, AS). Discussing sexual intimacy outside of the dyad could also be difficult. Some couples felt awkward initiating conversations with clinicians in order to seek help for improving sexual functioning, and this was sometimes not a topic of discussion approached by clinicians either. Across couples, both wives and husbands reported challenges in expressing emotions to each other, which some individuals compartmentalised (‘….but I think underlying all that is a potential level of anxiety and I know I’m better at boxing it off than (wife) is…’ Dyad 10, husband, 55–59, RP & EBR).

Couples who were able to communicate effectively with each other found this acted as a mutual self-support strategy which subsequently meant they often did not need to seek outside support:

‘If my wife wasn’t as close and didn’t talk to me then yes totally I would have more of a need to talk to others’ (Dyad 19, husband, 50-54, RP).

##### Intimacy matters

Disruptions occurred to some couples’ sexual relationships, which in turn could impact upon couples’ sense of ‘we-ness’. This was more common in men who experienced erectile dysfunction or loss of libido due to PCa or its treatment. Losing sexual intimacy at this period of their lives was regarded as a significant and distressing challenge for both men and women (‘I must admit I was a little bit depressed. It wasn’t ideal for a 49/50 year old or whatever to not get an erection’, Dyad 4, husband, 50–54, AS). One wife mourned the early loss of her love life:

‘I was mourning the end of our love life. I just felt almost like a widow, like I’d lost a husband, because I lost that intimacy or potentially lost that intimacy.[…] I still feel quite young in myself and I still feel very sexually active in myself so that upset me’ (Dyad 11, wife, 45–49, OT).

Couples married for shorter periods of time also reported challenges: ‘We’ve gone from having a very active sex life, we’ve not been married that long, to not really having any’ (Dyad 12, wife, 55–59, RP, EBR). When men physically withdrew from their wives, conflicts within the relationship could sometime result and therefore affect the couple unit (‘He kind of withdrew physically and of course then the frequency that we were intimate was less and then I get ratty because sometimes you just need a good shag right’ (Dyad 11, wife, 45–49, OT). Withdrawal was challenging for both members of the couple to face and these behaviours often led to couples perceiving their relationship transitioned from sexual to platonic:

‘You just feel well, oh I’m just living with this person and we’re sharing the same space, it’s more like student digs than anything else, it’s really really weird’ (Dyad 15, husband, 55–59, RP, EBR, HT).

A myriad of emotions were reported by couples in their engagement with sexual intimacy after treatment. Some couples reported disappointment and guilt over not being able to engage in sexual intimacy, which could contribute to a perception of premature ageing. The self-esteem of some of the men and women in the couple could also be harmed:

‘I feel tireder and older and part of that is it’s hard to feel as attractive when you don’t have the same sexual relationship with your husband anymore and that makes a difference to his sense of himself and to my sense of myself, and I get sad and I feel low about that sometimes’ (Dyad 12, wife, 55–59, RP, EBR).

In contrast, some couples were unconcerned by a lack of sexual activity because they focused on other parts of their relationship or viewed the man being alive as more important than sexual intimacy:

‘That lack of sexual function really isn’t an issue for us and it’s definitely not an issue for me, and (husband) tells me it’s not an issue for him. It is important in a relationship but it’s not as important as having him alive and here’ (Dyad 21, wife, 50–54, RP, EBR, HT).

Many couples recognised the importance of using sexual aids and/or medication to maintain sexual intimacy (‘It made the difference between having sex and not having sex basically. I think that’s the biggest impact certainly from a couple’s point of it’, Dyad 4, husband, 50–54, AS). Some couples that tried different sexual aids encountered challenges, including side effects of medication, lack of spontaneity in using aids, perceptions of devices as unappealing to use and look at, and sometimes, unsuccessful engagement with the device. Frustration ensued for one wife who wanted her husband to be more proactive in managing this part of their relationship and in persevering with treatments:

‘We’ve tried tablets, we’ve tried a pump, but he actually doesn’t want to use anything. I mean the pump seems like a really good idea but he really hated using that, and it wasn’t very successful. It was such a turn off. I find that a bit frustrating […] he doesn’t pursue the treatments for more than a few goes, and then we give up on any interventions really’ (Dyad 3, wife, 50-54, EBR, HT).

Couples often used humour and laughter about their situation when trying to adjust to transitions related to sexual changes in the relationship (‘We just had to have a laugh about it, when it wasn’t working properly. We sort of got through it like that’, Dyad 7, husband, 55–59, EBR, HT). Reassuring the man with PCa that they would work through any challenges to sexual intimacy was one way of support provided within the couple.

##### Distancing from unfamiliarity

A common behaviour employed within couples, particularly in which the man was receiving hormone treatment, was ‘distancing’ from one another. Men reported not feeling like the man they used to be before being on hormone treatment, which subsequently impacted upon the dynamics in the relationship with their wife and therefore how they connected with one another, (‘I felt like I was not me anymore, an imposter in my own body. I felt really out of place, I didn’t like going to the bathroom when my wife was in there, and I started using our other bathroom’, Dyad 26, husband, 60–65, HT, ST). In many cases, this sense of unfamiliarity in the relationship seemed to activate behaviours which caused difficulties in their adjustment to PCa and treatment. Wives whose husband had hormone treatment commonly reported their husband felt strange and unfamiliar to them, which, in some cases, made them feel less able to engage with husbands.

##### Mindset towards PCa

Another strategy couples engaged in to integrate and/or manage the impact of diagnosis and treatment of PCa was through their positivity and maintaining that positivity which helped couples cope with the PCa experience (‘We’re feeling quite positive and we’ve created that positivity in our lives’, Dyad 2, wife, < 45, AS). Some couples strove to make the best of a *bad* situation by making the most of their life. Many couples talked about ‘getting on with things’ as a mindset which helped them to adjust and detach from the impact of PCa on areas of their life as a couple and as individuals (‘We just got on with it, really, there were nothing else you can do really, is there?’, Dyad 2, husband, ≤ 45, AS). This approach also influenced the couples’ need for support and how they chose to manage their life. This, in turn, impacted upon their joint sense of how they saw themselves (i.e. evolution of their identity as a couple).

##### Work and finances: challenges, buffers, and new directions

The work lives of younger men and their partners were sometimes impacted upon, particularly for men who were experiencing side effects of treatment, incontinence, hot flushes, and fatigue:

‘I hit a really low point still suffering from incontinence, so I think it does knock your confidence dealing with the after effects really. I started getting anxiety and depression, so I was flying off the handle at work, and then I couldn’t face going to work in the morning… I had to take a little bit of time off work’ (Dyad 19, husband, 50–54, RP).

In some cases, their wives’ ability to work was also impacted by the PCa diagnosis and their own worries about their future as a couple (‘I was aware I was not performing as well. I was very forgetful, because I was in a position of responsibility and managing the team and I was aware that I needed to be cut a bit of slack maybe’, Dyad 3, wife, 50–54, EBR, HT).

The financial impact of PCa varied across couples. For many, financial buffers helped their situation, e.g. critical illness cover, personal independence payments (PIPs), (‘I was fortunate enough to have insurance for critical illness insurance so that pay saw me through the cash flow for the period that I was unable to work […]’, Dyad 22, husband, 55–59, RP). Couples in which one or both members were self-employed often found seeking financial welfare benefits difficult compared with those working for an employer. For some couples, in which the men took early retirement due to the impact of PCa, their wives sometimes reported feeling a financial burden due to one income in the household and therefore placed a pressure upon them to continue to work:

‘I'm still working because our finances have dropped considerably. You look forward to your futures when your children have grown up and sort of think when we retire but of course my husband has already retired and I’m still working, so I’m still talking when I retire, but there is the financial, I feel a financial burden’ (Dyad 6, wife, 55–59, RP & EBR).

Some couples were advised of financial support such as PIPs through charities. However, the benefits they received were not enough for them to enjoy activities which they used to, and so their social activities and travels abroad were curtailed. Guidance on where to access financial support was a commonly reported unmet need by couples (‘I think I needed somebody to talk to financially about where to go and stuff like that’, Dyad 19, husband, 50–54, RP).

For some couples, *distraction* was a helpful coping strategy from PCa. Couples often had busy lives, as the majority of couples were working which meant that cancer could be put to the back of their minds (‘I think there was so much going on in our lives, particularly with work, that (husband’s) cancer actually took a backseat, and sometimes you’d forget it until you thought about it because we were concentrating on life […]’, Dyad 11, wife, 45–49, OT).

A few women enjoyed having work as a distraction from thinking about PCa and a way for them to carry on life as normal (‘When I was working and nobody at work really knew because I could just kind of get away from it, I could just go and get on with working and that was good for me’, Dyad 25, wife, < 45, RP).

##### Re-evaluation of work life

Some men changed their job roles and working hours to accommodate the impact of treatment on their ability to work. Fatigue was a significant factor (particularly in men who were on hormone treatment or had radiotherapy), and some men reduced their working hours to facilitate this change (‘I have reduced my working hours. I was just finding it so hard because the hormone tablets do take it out of me, I do feel tired in the afternoon’, Dyad 7, husband, 55–59, EBR, HT).

For some men, the diagnosis of PCa made them re-evaluate their life and aspirations, and men nearing age of retirement often took early retirement. In one couple, the wife took retirement soon after her husband so they could spend more time together (‘Unfortunately (husband) was told that he wouldn’t be able to work anymore and I decided in that case I didn’t want to work, obviously we wanted to spend more time together’, Dyad 27, wife, 60–65, Chemo, HT, radium 223, ST). Re-evaluation of work life was also reported by some wives, through changed approaches to handling work situations and interacting with clients:

‘[…] I have a better understanding of what the PCa experience is like now…so I can sort of reassure my patients that I have that experience. […] and I think it’s bound to be helpful for my patient’s if I do have more insight’ (Dyad 6, wife, 55–59, RP & EBR).

##### Development of social connections and impact on social activities

A few couples reported varying changes in their social network with friends and community. Side effects of treatment (e.g. fatigue) impacted upon their ability to do things such as hobbies and therefore time spent together as a couple: ‘(Husband) and I don’t go out for meals and things like we used to because he doesn’t feel like having food’, Dyad 28, wife, 60–65, Chemo, HT, Radium 223).

Some couples found that friends were uninterested when discussing PCa with them, and a few couples mentioned feeling that cancer is a taboo subject in conversations with friends. Not knowing how to talk about or listen to experiences of PCa was perceived to be a barrier for some people in supporting these couples:

‘There was one friend and I did break down to her and she said “oh we'll get together”, and I haven’t seen her since, so you see people don’t want to hear it, or they don’t know how to talk about it, which is a shame’ (Dyad 17, wife, 50–54, RP).

Couples sometimes noticed the behaviour of friends changed towards them when topics about PCa were discussed. One man with advanced PCa experienced annoyance in people feeling sorry for him, when he felt well in himself:

‘Certainly one of the biggest things that I’ve noticed is other people are much more concerned about me being ill than I am, and it’s much more of a headache than anything else’ (Dyad 27, husband, 60–65, Chemo, HT, radium 223, ST).

##### Reassessing social engagements

A commonality amongst couples who were affected by an advanced diagnosis of PCa was their reduced engagement in social activities and/or lack of confidence in socialising with new people. In one couple, the wife’s social life was affected in that she did not want to socialise with friends for long periods of time, because she did not want to be away from her husband: ‘I’d carried on doing my Pilates and circuits but previously I would go out with my girlfriends quite a bit but I didn’t want to do that as much. If [husband] wasn’t doing anything I didn’t want him to be sat on his own and thinking about it’ (Dyad 7, wife, 60–65, EBR, HT).

However, one couple actually reported that they became more social post diagnosis, partly because they both became more involved in their religious community and this was used as a distraction from PCa:

‘I think we became way more social. I definitely say we became more social and more involved in our community as well. I mean who knows, maybe those were distractions’ (Dyad 1, wife, 45–49, RP, EBR & HT).

## Discussion

To our knowledge, this is the first study to explore in depth younger couples’ experiences of PCa. The study findings revealed that the diagnosis of PCa and side effects of treatment led couples to experience changes in core areas of their life (couples’ relationships, parental roles and family functioning (reported elsewhere [8]), work and finances, social connections, and activities). Couples may experience the impacts in different ways and manage these through using a range of strategies and engaging in different behaviours. Subsequently, dynamics within the relationship sometimes change which may impact upon the couple’s identity (see Fig. 1, online resource 4). Couple identity is a conceptualisation that views the couple relationship as an entity, rather than as two individuals [18]. As the findings of this study suggest, partner’s sense of shared identity can be changed in different ways due to PCa, including strengthening of the relational bond and shifts in established individual and dyadic identities, and potentially unexpected renegotiations in identities throughout survivorship. Even if couples’ satisfaction in the relationship remains the same, the experience of PCa can influence how these couples see themselves, understand, and navigate their relationship throughout survivorship.

This work builds on Manne and Badr’s [19] relationship intimacy model of couple’s psychosocial adaptation to cancer. This dyadic coping model offers a useful framework for considering our findings and implications for practice and shares similarities with some of the core themes in this study related to younger couples’ experiences of PCa. Manne and Badr’s [19] model focused on behaviours that contribute to the relationship in two ways: *relationship-enhancing* and *relationship-compromising* behaviours. Relationship-enhancing behaviours comprised reciprocal self-disclosure, partner responsiveness (feeling understood, cared for, acceptance by one’s partner), and relationship engagement (engaging in behaviours either aimed at sustaining or enhancing the relationship). Relationship-compromising behaviours were categorised into three broad categories of avoidance, criticism, and pressure-withdraw (pressuring partner to talk about cancer, leading to withdrawal). Some of these enhancing and compromising behaviours are congruent with the behaviours and strategies of younger couples in this study, for example, through withdrawal in instances of couples’ communication, disruption to sexual relationships, in their work life, dynamics within their family, and in social situations. It is well known that dyadic dysfunction is marked by withdrawal which may promote ineffective coping strategies that lead to lower relationship functioning [20, 21]. This was evident in couples in this study where maladaptive coping strategies such as withdrawal sometimes arose due to conflict between partners. Couples that take a ‘we-ness’ approach in managing the impact of PCa, for example, through communicating feelings to one another and reassuring each other about any concerns/worries, may provide a protective element of their relationship and in preserving a well-functioning relationship and developing greater resilience [22, 23]. Couples also employed strategies and behaviours to normalise the impacts of PCa on their parental role and dynamics within the family in ways that had controlled family life and the impact on the couple unit. Further discussion of this is reported elsewhere [8].

The extent to which ‘we-ness’ (couple identity) was reported by couples in this study varied and is highlighted through the mix of different engagement strategies and behaviours employed by members of the couple. A qualitative study that explored how couples cope with nutrition-related problems in advanced cancer [24] found that couples lean on different coping pathways (practical, emotional, or distant orientation). The study suggests that no one coping pathway is more superior to the other as they can all be adaptive, depending on the context. In this study on younger couples affected by PCa, the utilisation of ‘we-ness’ through humour and coming to terms with lack of intimacy seemed to be associated with a combination of different pathways which could lead to a more resilient way of coping. This is important in light of the findings of this study in which younger couples responded to the impact of various changes in their lives through the engagement of different strategies and behaviours. These were unique to the quality and dynamics of their relationship and what was helpful for them in their adjustment. For example, some couples used ‘distraction’ as a coping strategy, which may be considered maladaptive in the long term; however, for some couples this was a key strategy to help them adjust at that point in time.

For many couples, disruption to the sexual relationship was one of the major challenges they faced after a PCa diagnosis. Couples experiencing sexual dysfunction found difficulties in initiating or having discussions with healthcare professionals to seek help in this area. Difficulties in conversing about challenges in sexual relationships were also reported within the couples and subsequently led to withdrawal (a *relationship-compromising behaviour* as suggested by Manne and Badr’s [19] relationship intimacy model). Some couples experienced challenges in communicating about their true feelings to each other in general regarding their relationship, often to protect one another from further emotional distress, but this often hindered their adjustment and to some extent how they coped. Interventions which aid couple communication could therefore be helpful. A systematic review exploring the efficacy of existing couple-based interventions for patients with cancer and their partners [25] found promising figures on the uptake of telephone interventions for participants; however, some indicated a preference for some face-to-face contact.

Another core area for couples that was magnified due to the impact of PCa was their work and finances. Couples at this life stage may have higher financial responsibilities related to possible educational expenses of university-bound children and worries about retirement funds, and therefore further strain may be added to the relationship. For younger men who were active in the workplace prior to treatment, these changes were particularly difficult to adjust too. A large-scale UK population-based study that investigated factors associated with job loss and early retirement in men (age ≤ 60 years) diagnosed with PCa found that men with worse urinary and bowel symptoms had a greater likelihood of becoming unemployed [26]. The interview findings also offer a unique insight into the impact of PCa on wives/partner’s work lives and for members of the couple who are self-employed, something of which there is also minimal literature. For couples who were self-employed, knowing how to access financial support was a struggle. There is literature suggesting that whilst financial concerns can cause psychological distress and impact upon family wellbeing, these needs often go undetected by clinical staff [27, 28]. Couple’s pre-occupation with cancer and possible reluctance in communicating about negative impacts of cancer means that financial planning may be delayed until later on. It may be helpful for healthcare advisors to prompt or refer couples who are having radical treatment to examine insurance policies, alerting them to employment rights and basic information about benefits and grants so as not to add financial and existential worries to the psycho-emotional burden of the family [29, 30].

Few couple intervention studies have been undertaken in the UK. McCaughan et al. [30] conducted a feasibility randomised controlled trial called the CONNECT programme that was designed to improve participants’ belief in their capabilities to manage PCa as a team, through addressing symptom and uncertainty management, sexual dysfunction, positive thinking, and healthy lifestyle. A qualitative exploration of couples’ perceptions of the CONNECT programme [31] found that participants valued the opportunity to share coping strategies and listen to others’ accounts to receive reassurance and validation of their own experiences. Therefore, the opportunity to talk to other men and their partners affected by PCa who are of a similar age and experiences through a type of buddying system may be helpful. Furthermore, a similar programme with a focus on addressing some of the issues that were magnified for these younger couples and ways to address relationship-enhancing strategies and behaviours may be useful. Previous research indicates that the quality of a couple’s relationships plays an important role in helping them maintain their physical and psychological wellbeing [32]. Therefore, it is important that the focus of couple interventions be aimed at maintaining a strong partnership to buffer against the challenges they may face and strategies and behaviours to employ to manage/integrate the impact of PCa on their lives.

## Study limitations

Younger couples interviewed in this study were 2 to 5 years post diagnosis of PCa, so recall bias is possible. The sample was not diverse, and the experiences of couples from Black, Asian, and minority ethnic (BAME) groups and same sex couples are not reflected. Lastly, the majority of couples had been married for over 20 years. The length of time couples had been together may impact upon the level of adjustment in couples and therefore their experiences and needs.

## Clinical implications

Supportive guidance programmes that facilitate couple adjustment following a PCa diagnosis should be developed to foster relationship-enhancing strategies and behaviours, particularly in communication and sexual intimacy. Strategies are needed to raise awareness of existing resources/tools amongst healthcare professionals that could support discussing sexual intimacy with couples. Tailored information to couples within this age group, specifically online support, should also be available through written materials or mobile application to provide information on financial guidance and other self-management strategies for younger couples. A ‘buddying system’ which connects younger couples affected by PCa together may also be helpful.

## Conclusions

Younger couples experienced changes in their relationships, work and finances, parenthood and family functioning, and social connections and activities. Couples engaged in various strategies and behaviours which influenced their adjustment as a couple. The strategies/behaviours couples engaged in to manage the impacts of these changes on their lives and relationship contributed to the evolution of their couple identity. Regardless of the stage of cancer, adaptation to cancer from a couples’ perspective was dependent on how well the couple integrated cancer into their lives and managed these impacts as a couple. It is important that younger couples have access to services or resources which can address their unmet needs and help them move forward as a couple.

## Electronic supplementary material

ESM 1 (PDF 716 kb)

ESM 2 (PDF 311 kb)

ESM 3 (PDF 107 kb)

ESM 4 (PDF 638 kb)
