# Supplementary material for: The experiences and needs of couples affected by prostate cancer aged 65 and under: a qualitative study
Source: J Cancer Surviv. 2020 Sep 24;15(2):358–66. doi: 10.1007/s11764-020-00936-1 (PMC7966139; doi:10.1007/s11764-020-00936-1)
Supplement: Supplementary file 1 — (PDF 716 kb) [file 11764_2020_936_MOESM1_ESM.pdf]

Online Resource 1- Consolidated criteria for reporting qualitative studies (COREQ)

The experiences and needs of couples affected by prostate cancer aged 65 and under; a qualitative study.

Journal of Cancer Survivorship

Nicole Collaço[1,2*], Richard Wagland[1], Obrey Alexis[2], Anna Gavin[3], Adam Glaser[4], Eila K Watson[2]

[1] Faculty of Health Sciences, University of Southampton, S017 1BJ

[2]Faculty of Health and Life Sciences, Oxford Brookes University, Jack Straws Lane, Oxford, OX3 0FL

[3] Northern Ireland Cancer Registry Centre for Public Health, School of Medicine, Dentistry and Biomedical Sciences, Queen's University, Belfast, BT12 6BA

[4] Leeds Institute of Cancer and Pathology, Faculty of Medicine and Health, University of Leeds, Worsley Building, Leeds, LS2 9NL

*Correspondence to:

Nicole Collaço[1]

University of Southampton

Email: n.b.collaco@soton.ac.uk

Manuscript: The experiences and needs of couples affected by prostate cancer aged 65 and under; a qualitative study.

Consolidated criteria for reporting qualitative studies (COREQ): 32-item checklist

Developed from:

Tong A, Sainsbury P, Craig J. Consolidated criteria for reporting qualitative research (COREQ): a 32-item checklist for interviews and focus groups. *International Journal for Quality in Health Care*. 2007. Volume 19, Number 6: pp. 349 – 357

No. Item	Guide questions/description	Reported on Page #
**Domain 1: Research team and reflexivity**		
Personal Characteristics		
1. Interviewer/facilitator	Which author/s conducted the interview or focus group?	Nicole Collaco, Page 5
2. Credentials	What were the researcher's credentials? E.g. PhD, MD	PhD (Not required to be stated by journal)
3. Occupation	What was their occupation at the time of the study?	PhD Student Page 5
4. Gender	Was the researcher male or female?	Female. Page 5
5. Experience and training	What experience or training did the researcher have?	Page 5
Relationship with participants		
6. Relationship established	Was a relationship established prior to study commencement?	No Page 5
7. Participant knowledge of the interviewer	What did the participants know about the researcher? e.g. personal goals, reasons for doing the research	Participant information sheet and consent form Methods Page 5 under *Recruitment and Data Collection*
8. Interviewer characteristics	What characteristics were reported about the inter viewer/facilitator? e.g. Bias, assumptions, reasons and interests in the research topic	Reported on page 4-5 under *Recruitment and Data Collection.*

**Domain 2: study design**		
Theoretical framework		
9. Methodological orientation and Theory	What methodological orientation was stated to underpin the study? e.g. grounded theory, discourse analysis, ethnography, phenomenology, content analysis	Methods- Data analysis<br>Page 5
Participant selection		
10. Sampling	How were participants selected? e.g. purposive, convenience, consecutive, snowball	Methods under *Recruitment and Data Collection*<br>Page 4
11. Method of approach	How were participants approached? e.g. face-to-face, telephone, mail, email	Methods<br>Page 5 under *Interview Procedures*
12. Sample size	How many participants were in the study?	Results<br>Page 6
13. Non-participation	How many people refused to participate or dropped out? Reasons?	Results- *study sample*<br>Page 6
Setting		
14. Setting of data collection	Where was the data collected? e.g. home, clinic, workplace	Page 5 under *Interview procedures*
15. Presence of non-participants	Was anyone else present besides the participants and researchers?	No.<br>Page 5 under *Interview procedures*
16. Description of sample	What are the important characteristics of the sample? e.g. demographic data, date	Table 1. (see online resource 3).<br>Referred to on page 6
Data collection		
17. Interview guide	Were questions, prompts, guides provided by the authors? Was it pilot tested?	Methods, under *interview procedures*<br>Page 5
18. Repeat interviews	Were repeat inter views carried out? If yes, how many?	No.<br>Page 5 under *Interview procedures*
19. Audio/visual recording	Did the research use audio or visual recording to collect the data?	Methods<br>Page 4

20. Field notes	Were field notes made during and/or after the inter view or focus group?	Analysis<br>Page 5
21. Duration	What was the duration of the inter views or focus group?	Page 5 under *Interview procedures*
22. Data saturation	Was data saturation discussed?	Analysis<br>Page 5
23. Transcripts returned	Were transcripts returned to participants for comment and/or correction?	No.<br>Page 5 under *Interview procedures*
**Domain 3: analysis and findings**		
Data analysis		
24. Number of data coders	How many data coders coded the data?	Four (NC, EW, RW, OA)<br>Data Analysis<br>Page 5
25. Description of the coding tree	Did authors provide a description of the coding tree?	Page 5 – under Data analysis.<br>See also online resource 2.
26. Derivation of themes	Were themes identified in advance or derived from the data?	Page 5 – under Data analysis.<br>See also online resource 2.
27. Software	What software, if applicable, was used to manage the data?	Data Analysis.<br>Page 5.
28. Participant checking	Did participants provide feedback on the findings?	No.<br>Page 5 under *Interview procedures*
Reporting		
29. Quotations presented	Were participant quotations presented to illustrate the themes/findings? Was each quotation identified? e.g. participant number	Results<br>Pages 6-14
30. Data and findings consistent	Was there consistency between the data presented and the findings?	Yes.<br>Page 6-14
31. Clarity of major themes	Were major themes clearly presented in the findings?	Yes.<br>From page 6 to 14
32. Clarity of minor themes	Is there a description of diverse cases or discussion of minor themes?	Discussion of major and minor themes<br>From pages 14-17
