# Supplementary material for: The experiences and needs of couples affected by prostate cancer aged 65 and under: a qualitative study
Source: J Cancer Surviv. 2020 Sep 24;15(2):358–66. doi: 10.1007/s11764-020-00936-1 (PMC7966139; doi:10.1007/s11764-020-00936-1)
Supplement: Supplementary file 2 — (PDF 311 kb) [file 11764_2020_936_MOESM2_ESM.pdf]

Online Resource 2: Section of theme development with summaries from Framework Matrix

The experiences and needs of couples affected by prostate cancer aged 65 and under; a qualitative study.

Journal of Cancer Survivorship

Nicole Collaço[1,2*], Richard Wagland[1], Obrey Alexis[2], Anna Gavin[3], Adam Glaser[4], Eila K Watson[2]

[1] Faculty of Health Sciences, University of Southampton, S017 1BJ

[2]Faculty of Health and Life Sciences, Oxford Brookes University, Jack Straws Lane, Oxford, OX3 0FL

[3] Northern Ireland Cancer Registry School of Medicine, Dentistry and Biomedical Sciences, Centre for Public Health, Queen's University, Belfast, BT12 6BA

[4] Leeds Institute of Cancer and Pathology, Faculty of Medicine and Health, University of Leeds, Worsley Building, Leeds, LS2 9NL

*Correspondence to:

Nicole Collaço[1]

University of Southampton

Email: n.b.collaco@soton.ac.uk

Online resource 2: Section of theme development with summaries from Framework

	Ability to work	Work adjustments	Reassessing the meaning of work	Financial impact	Support from work
Dyad 20<br>Active surveillance<br>**M: 53 YO**<br>**P: 30 YO**<br>**Married: 4 months**<br>**Children: 22 months**	Whilst he felt that there was no impact to his work life, she struggled to concentrate at work as she had a lot going on in her mind with different stressors contributing to her inability to concentrate, which were to do with the relationship being new, possibility of not having children, and his diagnosis of PCa.			No financial implications from experience of PCa even though time off was taken for attending appointments and biopsies.	
Dyad 3<br>EBR, HT<br>**M: 55 YO**<br>**P: 54 YO**<br>**Married: 32 years**<br>**Children: 3 (in 20's)**	She was thought she was not performing as well at work as she was forgetful and because she was in a position of managerial responsibility she felt she had to tell her colleagues at work so they were aware of the reason for her changed behaviour.<br><br>Side effects of treatment didn't affect his ability to work and did not need to take time off work other than to attend radiotherapy sessions.	She reduced her hours at work to support husband but also son whose partner had recently had a baby.<br><br>He doesn't travel as much as he used to since his diagnosis of PCa.	After his sister died young from breast cancer, it hit him hard that he didn't want to work till he was 60/65, and because his work pension was generous, it made it financially possible to leave at a younger age, so when he was diagnosed with PCa, his immediate decision was to give up work and wife did the same so that they could make the most of the time they had left together, if it was limited. He hasn't regretted stopping work. Wife also decided to retire young with husband so they could spend more time together and make the most of their life together.	There was no financial impact- they both didn't take time off from work, and she feels it drove him to work harder to give them financial security in coming years.<br><br>In the early stages post diagnosis, he had a meeting with his financial advisor on pensions as he and his wife wandered about the longevity of his career which impacted on his attitude to work, as he never let go of his desire to work and be successful in his efforts, but he was also considering what other options he had if he didn't want to continue with work.	She found her work were understanding and supportive, and was able to take of time she needed to attend appointments.
Dyad 5<br>Chemo & HT<br>(ADVANCED)<br>**M: 52 YO**<br>**P: 45 YO**<br>**Married: 23 years**<br>**Children: 21 YO**	He had work plans to work for certain periods of time but was unable to do so when he got diagnosed with PCa.	He took medical retirement as he was unable to work post treatment.		They sought support for finances through charity support, who made them aware of PIPs. Initially they were able to pay some of the mortgage off from medical retirement money, however the benefits they receive isn't enough for them to do extra things which they used to do, so that has impacted on their social activities. He felt that day to day living was causing financial burden. They couldn't afford to go on holiday because of the cost of medical insurance.	His employers were supportive, and allowed him to take off as much time as he needed, but eventually he had to quit because of his treatments and the impact.

RELATIONSHIPS | FAMILY | CHILDREN | SOCIAL | **WORK & FINANCES** | +
