# Supplementary material for: The experiences and needs of couples affected by prostate cancer aged 65 and under: a qualitative study
Source: J Cancer Surviv. 2020 Sep 24;15(2):358–66. doi: 10.1007/s11764-020-00936-1 (PMC7966139; doi:10.1007/s11764-020-00936-1)
Supplement: Supplementary file 3 — (PDF 107 kb) [file 11764_2020_936_MOESM3_ESM.pdf]

Online Resource 3- Table 1: Demographic and diagnosis characteristics of interviewed couples

The experiences and needs of couples affected by prostate cancer aged 65 and under; a qualitative study.

Journal of Cancer Survivorship

Nicole Collaço[1,2*], Richard Wagland[1], Obrey Alexis[2], Anna Gavin[3], Adam Glaser[4], Eila K Watson[2]

[1] Faculty of Health Sciences, University of Southampton, S017 1BJ

[2]Faculty of Health and Life Sciences, Oxford Brookes University, Jack Straws Lane, Oxford, OX3 0FL

[3] Northern Ireland Cancer Registry Centre for Public Health, School of Medicine, Dentistry and Biomedical Sciences, Queen's University, Belfast, BT12 6BA

[4] Leeds Institute of Cancer and Pathology, Faculty of Medicine and Health, University of Leeds, Worsley Building, Leeds, LS2 9NL

*Correspondence to:

Nicole Collaço[1]

University of Southampton

Email: n.b.collaco@soton.ac.uk

Table 1: Demographic and diagnosis characteristics of interviewed couples

Characteristic	Number (%)
**Ethnicity**	
White British	27 (96%)
Other	1 (4%)
**Ages**	
**Men**	Mean- 54.7 years Range- (42-65) SD- 5.4
60-65	4 (14%)
55-59	10 (36%)
50-54	12 (43%)
≤45	2 (7%)
**Women**	Mean- 52.5 years Range- (33-67) SD- 8.8
≥65	3 (11%)
60-65	3 (11%)
55-59	6 (21%)
50-54	10 (36%)
45-49	2 (7%)
<45	4 (14%)
**Length of Marriage/partnership**	
≤10	7 (25%)
11-20	5 (18%)
21-30	9 (32%)
31-40	6 (21%)
>40	1 (4%)
**Couples with children in the following age categories (ages)**	
Couples with no children	1 (4%)
Couples with non-adult children (< 18 years)	8 (28%)
Couples with young adult children (≥ 18 years)	12 (43%)
Couples who have both non-adult & young adult children	3 (11%)
Couples with adult children (≥ 30 years)	4 (14%)
**Employment**	
**Men**	
Full time/Part time	14 (50%)
Self-Employed	7 (25%)

Retired	7 (25%)
**Women**	
Full time/Part time	21 (75%)
Self-Employed	1 (4%)
Unemployed	1 (4%)
Retired	5 (17%)
**Mean Time Since Diagnosis (years)**	3.2
**Treatment**	
Surgery alone (RP)	6 (21%)
Surgery & radiotherapy (EBR)	3 (11%)
Surgery, radiotherapy & hormone therapy (HT)	3 (11%)
Radiotherapy & hormone therapy	4 (14%)
Active surveillance (AS)	7 (25%)
Hormone therapy & adjuvant therapy (chemotherapy, radium 223, Enzalutamide/Abiraterone)	4 (14%)
Other treatment (OT)	1 (4%)
STAMPEDE Trial (ST)[1]	3 (11%)
**Stage of PCa**	
Stage 1	7 (25%)
Stage 2	7 (25%)
Stage 3	8 (29%)
Stage 4	5 (18%)
Not known	1 (3%)
**Healthcare**	
Private	6 (21%)
NHS	22 (79%)
**Family history of PCa**	10 (36%)

[1] STAMPEDE (Systemic Therapy in Advancing or Metastatic Prostate Cancer: Evaluation of Drug Efficacy- A large trial that aimed to assess new treatment approaches for locally advanced or metastatic PCa.
