# Supplementary material for: The experiences and needs of couples affected by prostate cancer aged 65 and under: a qualitative study
Source: J Cancer Surviv. 2020 Sep 24;15(2):358–66. doi: 10.1007/s11764-020-00936-1 (PMC7966139; doi:10.1007/s11764-020-00936-1)

Online Resource 4- Figure 1- Conceptualisation of Findings

The experiences and needs of couples affected by prostate cancer aged 65 and under; a qualitative study.

Journal of Cancer Survivorship

Nicole Collaço[1,2*], Richard Wagland[1], Obrey Alexis[2], Anna Gavin[3], Adam Glaser[4], Eila K Watson[2]

[1] Faculty of Health Sciences, University of Southampton, S017 1BJ

[2]Faculty of Health and Life Sciences, Oxford Brookes University, Jack Straws Lane, Oxford, OX3 0FL

[3] Northern Ireland Cancer Registry Centre for Public Health, School of Medicine, Dentistry and Biomedical Sciences, Queen's University, Belfast, BT12 6BA

[4] Leeds Institute of Cancer and Pathology, Faculty of Medicine and Health, University of Leeds, Worsley Building, Leeds, LS2 9NL

*Correspondence to:

Nicole Collaço[1]

University of Southampton

Email: n.b.collaco@soton.ac.uk

Figure 1- Conceptualisation of Findings

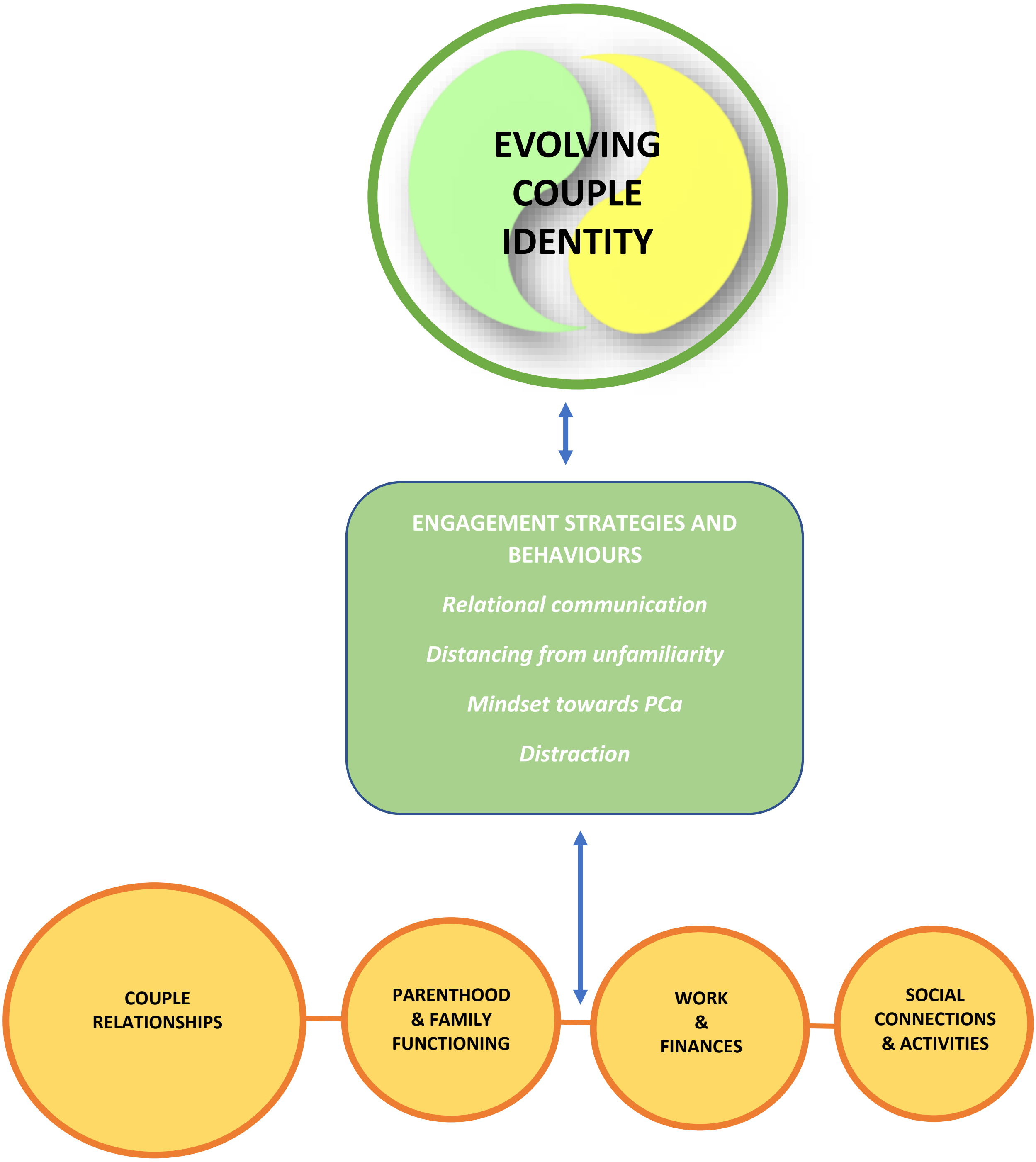

Supplement: Supplementary file 4 — (PDF 638 kb) [file 11764_2020_936_MOESM4_ESM.pdf]
